# The effects and costs of laparoscopic versus abdominal myomectomy in patients with uterine fibroids: a systematic review and meta-analysis

**DOI:** 10.1186/s12893-020-00703-0

**Published:** 2020-03-20

**Authors:** Ruixin Chen, Zhiying Su, Lingling Yang, Luping Xin, Xiaodong Yuan, Yanlong Wang

**Affiliations:** Department of Gynecology, Women and Children’s Hospital, School of Medicine, Xiamen University, NO.10 Zhenhai Road, Siming District, Xiamen, China

**Keywords:** Laparoscopy, Myomectomy, Uterine fibroids

## Abstract

**Background:**

Abdominal myomectomy (AM) and laparoscopic myomectomy (LM) are commonly see surgery for the uterine fibroids, several randomized controlled trials (RCTs) have compared the role of AM and LM, the results remained inconsistent. Therefore, we attempted this meta-analysis to analyze the role of LM versus AM in patients with uterine fibroids.

**Methods:**

We searched PubMed et al. databases from inception date to July 31, 2019 for RCTs that compared LM versus AM in patients with uterine fibroids. Two authors independently screened the studies and extracted data from the published articles. Summary odd ratios(OR) or mean differences(MD) with 95% confidence intervals(CI) were calculated for each outcome by means of fixed- or random-effects model.

**Results:**

Twelve RCTs with a total of 1783 patients were identified, with 887 patients for and 897 patients for AM. Compared with AM, LM could significantly decrease the blood loss (OR = − 29.78, 95% CI -57.62– − 0.95), shorten the duration of postoperative ileus (OR = − 10.91, 95% CI -18.72– − 3.11), reduce the length of hospital stay (OR = − 1.57, 95% CI -2.05– − 1.08), but LM was associated with longer duration of operation (OR = 16.10, 95% CI 6.52–25.67) and higher medical cost (OR = 17.61, 95% CI 7.34–27.88).

**Conclusions:**

LM seems to be a better choice for patients with uterine fibroids, more related studies are needed to identify the role of LM and AM for the treatment of uterine fibroids.

## Background

Uterine fibroids are the most common benign tumors, and the prevalence of uterine fibroids in premenopausal women is reported to be between 20 and 40% [1, 2]. Although uterine fibroids as are often asymptomatic, it can cause abnormal uterine bleeding, infertility, pelvic pain and even miscarriage [3]. Uterine myomectomy is desirable for women of childbearing age who wish to maintain potential fertility, especially when uterine fibroids have symptoms that abnormal uterine bleeding or pain, or asymptomatic but rapidly growing and causing recurrent miscarriage [4]. Uterine myomectomy by laparoscopy or laparotomy are usually performed based on the location, size, number of uterine fibroids and surgeon experience [5, 6].

Abdominal myomectomy (AM) is a kind of classic surgeries for uterine fibroids, meanwhile laparoscopic myomectomy (LM) has also been widely used in clinical practice because of its advantages of mild trauma, less complications, and rapid postoperative recovery [7–9]. At present, there are many related studies on the advantages and disadvantages of AM and LM in patients with uterine fibroids at home and abroad. However, most of them are single-centered, and the sample sizes are small, further objective and economic evaluations on the role of AM and LM are needed. Therefore, we attempted to conduct this systematic review and meta-analysis to evaluate the effects and costs of LM and AM in patients with uterine fibroids as follows.

## Methods

This present meta-analysis was reported in accordance with the criteria of Preferred Reporting Items for Systematic Reviews and Meta-analysis (PRISMA) [10].

### Search strategy

Two reviewers independently conducted the systematic searches of related literature. The databases searched included Medline, PubMed, EMBASE, Cochrane Library, China National Knowledge Infrastructure (CNKI) and Wanfang Database, China biomedical literature database (CBM). The literature search of each database was conducted up to July 31, 2019. Language restrictions on studies published in English and Chinese were imposed. The randomized controlled trials (RCTs) on the role of AM and LM were identified. The following terms and their combinations were searched in related databases: uterine myoma OR uterine fibroids OR leiomyomas, laparoscopic surgery OR laparotomy OR abdominal myomectomy or open surgery. The reference lists of previously published reviews were also reviewed and manually searched. Potential unpublished studies that may be eligible were also searched from the WHO International Clinical Trial Registry. Any disagreements were discussed with a third reviewer to reach a consensus.

### Inclusion and exclusion criteria

The study was selected on the basis of the first screening of the identified title or abstract and the second full-text examination. The included studies must meet following inclusion criteria: (1) RCT design; (2) compared the effects of LM and AM in patients with Uterine fibroids; (3) the details of LM and AM procedure were reported; (4) related study results were reported. Studies were excluded from this meta-analysis if (1) the outcomes of interest were not clearly reported; (2) extracting the related data from the published results is impossible; (3) Considerable overlaps between the authors, research centers among the published literature.

### Data extraction

We used a standardised data collection form to extract key information. Any discrepancies in the extraction process were resolved by consensus. We also attempted to contact authors to obtain additional data or to clarify data of missing details. Two reviewers independently extracted the following information: first author, year of publication, study location, patient population, details of LM and AM, main outcomes and study results. The following main outcome measurements were also extracted and analysed in this present meta-analysis: duration of operation, the blood loss, the length of hospital stay, the duration of postoperative ileus, and the cost of LM and AM treatment.

### Quality assessment

The Cochrane Collaboration’s risk of bias tool [11] was used by two reviewers independently to evaluate the methodological quality and risk of bias of the included RCTs; any disagreements were resolved by discussion and consensus. This tool was also utilised to examine and measure seven specific domains: sequence generation, allocation concealment, blinding of participants and personnel, blinding of outcome assessment, incomplete outcome data, selective outcome reporting and other issues. Each domain could be classified as low risk of bias, high risk of bias or unclear risk of bias according to the judgement criteria.

### Data analysis

All statistical analyses were performed with RevMan 5.3 software. Data were used as input and double-checked by two reviewers. Data syntheses and interpretations were also performed by two authors to ensure the accuracy of the results. Binary outcomes were presented as Mantel–Haenszel-style odds ratios with 95% confidence intervals. Continuous outcomes were reported as mean differences (MDs). A fixed-effect model was adopted in cases of homogeneity (*P*-value of χ^2^ test > 0.10 and *I*^2^ < 50%), whereas a random-effect model was used in cases of obvious heterogeneity (P-value of χ^2^ test > 0.10 and *I*^2^ ≥ 50%) [12]. Publication bias was evaluated by using funnel plots, and asymmetry was assessed by conducting Egger regression test. For funnel plot asymmetry, *P* < 0.1 was considered significant.

## Results

**Fig. 1:**
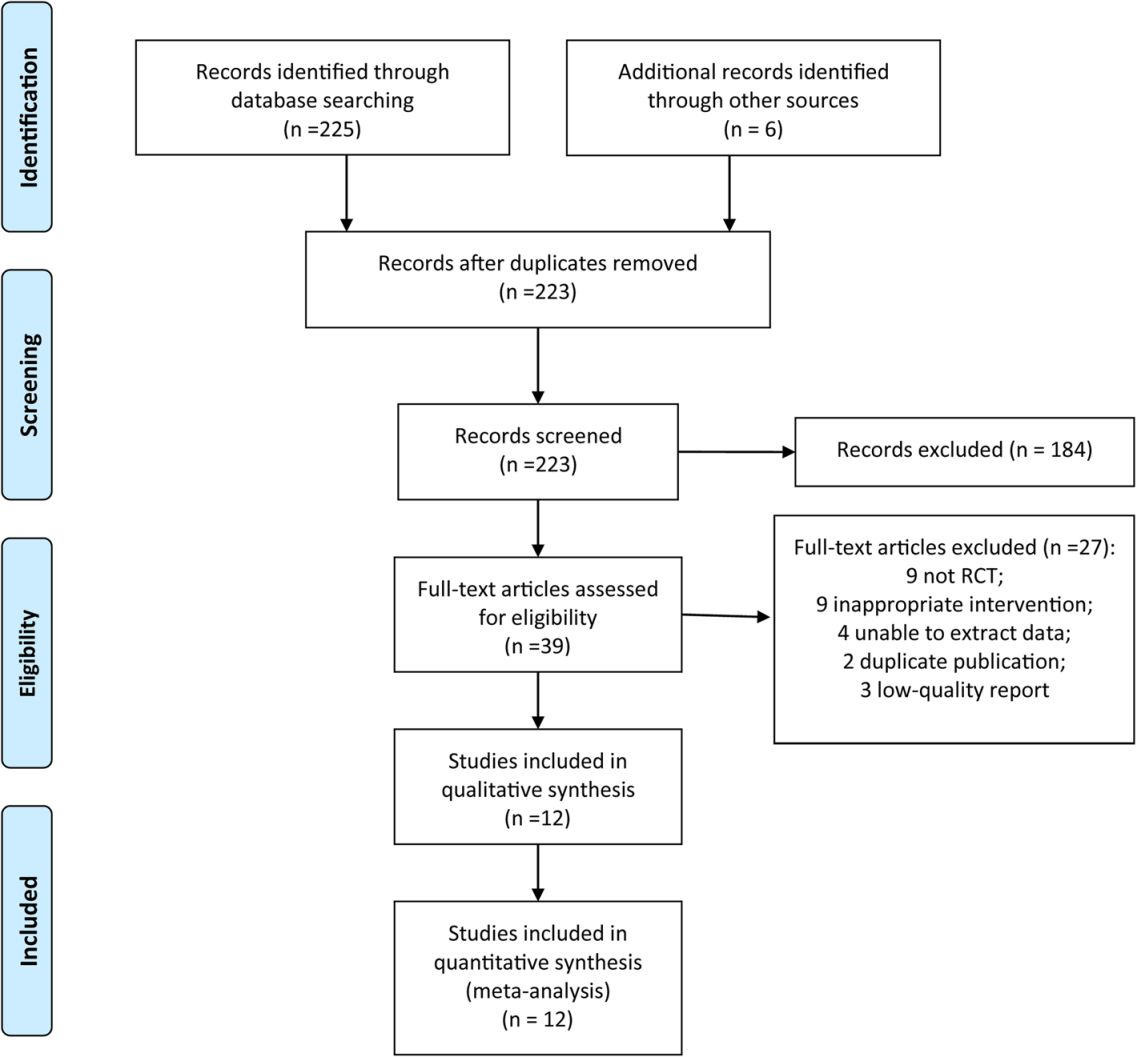
Flow chart of study selection

The initial literature search yielded 231studies. The number of duplicated articles removed was 223. Furthermore, a total of 184 studies were excluded after screening the titles and abstracts. Thirty nine studies were reviewed for eligibility by scrutinizing full-text articles. Eventually, 12 eligible RCTs [13–24] were included in this meta-analysis. The PRISMA flowchart is showed in Fig. 1.

### The characteristics of included RCTs

The basic characteristics of the 12 included RCTs are presented in Table 1. These 12 RCTs included a total study population of 1783 randomized participants, with 887 LM patients and 897 AM patients. The sample sizes ranged from 40 to 384 patients. Six studies [13, 14, 16, 18–20] were conducted in Italy and the remaining six RCTs [15, 17, 21–24] were conducted in China. The mean age of included patients varied from 28 to 50 years old, and the follow-up period differed from 1 to more than 12 months. The results from most studies supported the use of LM in patients with uterine fibroids.

**Table 1:** The characteristics of included RCTs

Studies	Countries	Participants (LM/AM)	Mean age (LM/AM)	Follow-up (month)	Findings
Alessandri 2006 [13]	Italy	74/74	37.5 ± 4.5/38.4 ± 64.9	6	Laparoscopic myomectomy may offer the benefits of lower postoperative analgesic use and faster postoperative recovery.
Cicinelli 2009 [14]	Italy	40/40	32.1 ± 8.5/34.3 ± 9.3	6	Laparoscopic myomectomy is a suitable in women with 1 to 3 myomas.
Ding 2017 [15]	China	60/60	37.52 ± 6.21/ 7.44 ± 7.18	NA	Laparoscopic uterine myoma decollement provides shorter operation time, less intraoperative bleeding volume, quicker recovery and higher safety in patients.
Fanfani 2005 [16]	Italy	93/120	34.4(26–40)/33.6(24–39)	1	Myomectomy by minilaparotomy can be considered a minimally invasive alternative to laparoscopy in the surgical management of intramural and subserosal myomas.
Li 2011 [17]	China	120/120	40.0 ± 10.0/50.0 + 10.0	NA	The cost-effective effect of laparoscopic uterine fibroids excision is better than traditional abdominal myomectomy.
Mais 1995 [18]	Italy	20/20	34.3 ± 6.3/33.8 ± 6/7	NA	Laparoscopic myomectomy may offer the benefits of lower postoperative pain and shorter recovery time in comparison with laparotomy.
Palomba 2007 [19]	Italy	68/68	28 (21–36)/28 (22–38)	12	A careful evaluation of the dimensions and localizations of fibroids are needed to address to the right choice to the best approach.
Seracchioli 2000 [20]	Italy	66/65	34.00 ± 4.11/33.97 ± 4.79	≥12	LM can be performed in a great number of cases even in the presence of very large myomata
Wang 2010 [21]	China	38/34	37.5(30–51)/38.5(31–50)	NA	LM has a significant effect on the treatment of large or multiple uterine fibroids, with the advantages of small trauma, short hospital stay, less complications, and quick recovery.
Wang 2011 [22]	China	194/190	37.6 ± 7.3/36.7 ± 8.2	NA	gasless laparoscopy is safe and reliable in myomectomy.
Yang 2011 [23]	China	31/30	38.3/36.8	NA	Gasless laparoscopic multiple myomectomy is a good minimally invasive procedure.
Zhang 2012 [24]	China	82/76	36.6 ± 5.2/35.8 ± 6.1	NA	Compared with AM, LM has the advantages of less trauma, less bleeding, faster recovery and shorter hospital stay. It has obvious advantages under certain conditions, but it cannot completely replace AM.

### Quality evaluation

**Fig. 2:**
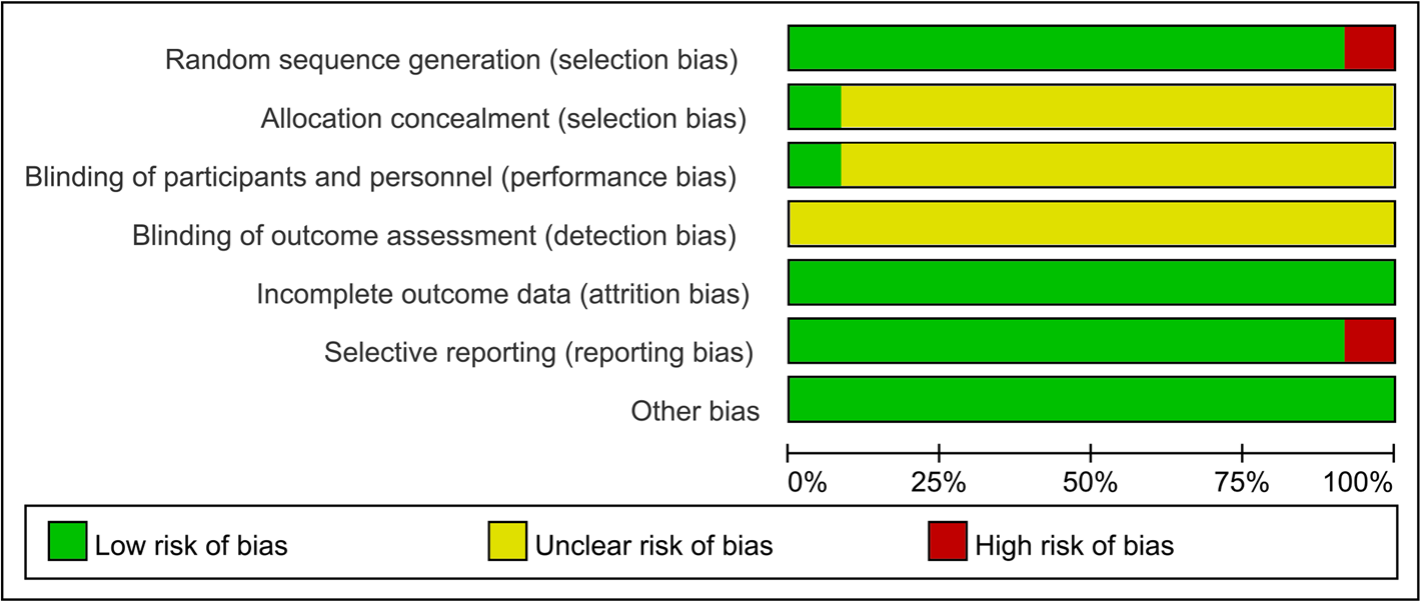
Risk of bias graph

**Fig. 3:**
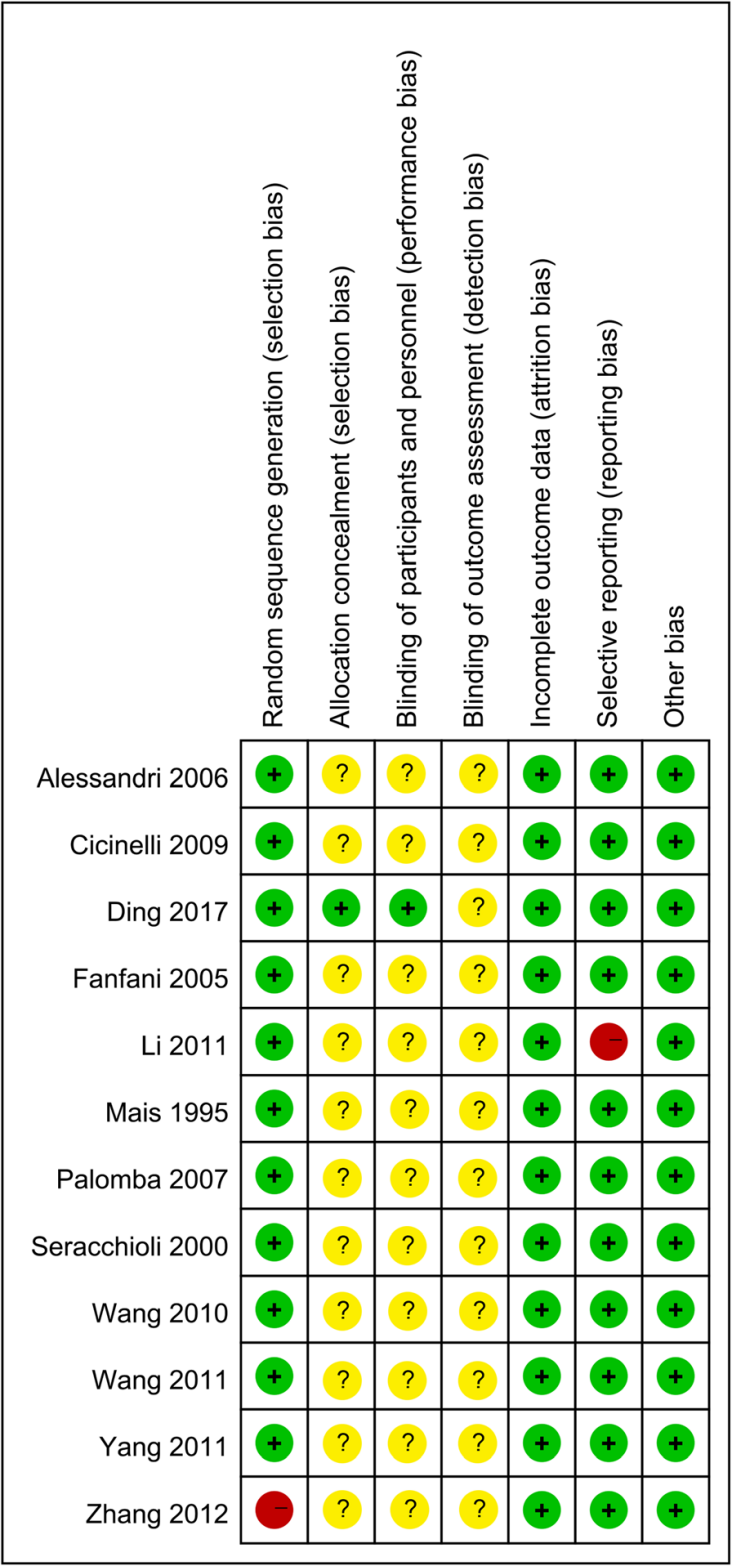
Risk of bias summary

The results of the methodological quality evaluation are presented in the Figs. 2 and 3. Following strict judgments of each included RCT according to the Cochrane handbook, although all of the 12 included RCTs mentioned randomization, no RCT provided a detailed description of the methods used to produce a random sequence, and one study [24] reported an incorrect randomization method. Most of the included RCTs didn’t report allocation blinding or the personnel blinding, only one study [15] reported blind design on allocation and personnel. For the blinding of outcome assessment, all included studies didn’t report the related information. In addition, one study [17] only reported cost of LM and AM, and no other outcomes were presented. As such, this study displayed a high risk of bias in terms of incomplete outcome data and selective reporting. No other significant biases amongst the included RCTs were found.

### Outcomes

#### Blood loss

**Fig. 4:**
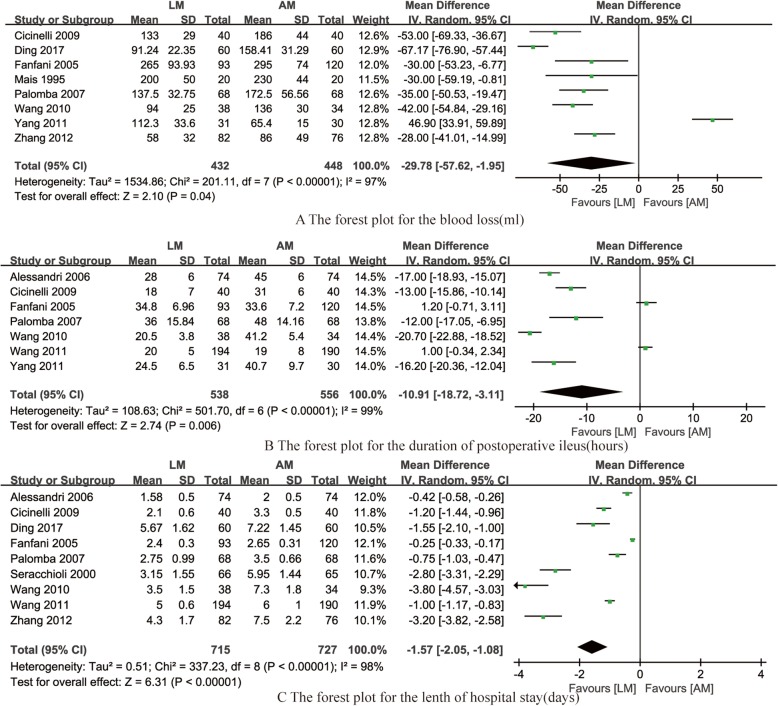
Forest plot for synthesized outcomes

Eight studies [14–16, 18, 19, 21, 23, 24] reported the blood loss during the LM and AM, the pooled data from the eight RCTs revealed that LM could significantly decrease the blood loss compared with AM (OR = − 29.78, 95% CI -57.62– -0.95, *P* = 0.05, *I*^*2*^ = 95%; Fig. 4a).

#### The duration of postoperative ileus

Seven studies [13, 14, 16, 19, 21–23] reported the duration of postoperative ileus, the pooled data from the eight RCTs revealed that LM could significantly shorten the duration of postoperative ileus compared with AM (OR = − 10.91, 95% CI -18.72– -3.11, *P* = 0.006, *I*^*2*^ = 99%; Fig. 4b).

#### The length of hospital stay

Nine studies [13–16, 19–22, 24] reported the length of hospital stay, the pooled data from the nine RCTs revealed that LM could significantly reduce the length of hospital stay compared with AM (OR = − 1.57, 95% CI -2.05– -1.08, *P* < 0.001, *I*^*2*^ = 98%; Fig. 4c).

#### The duration of operation

**Fig. 5:**
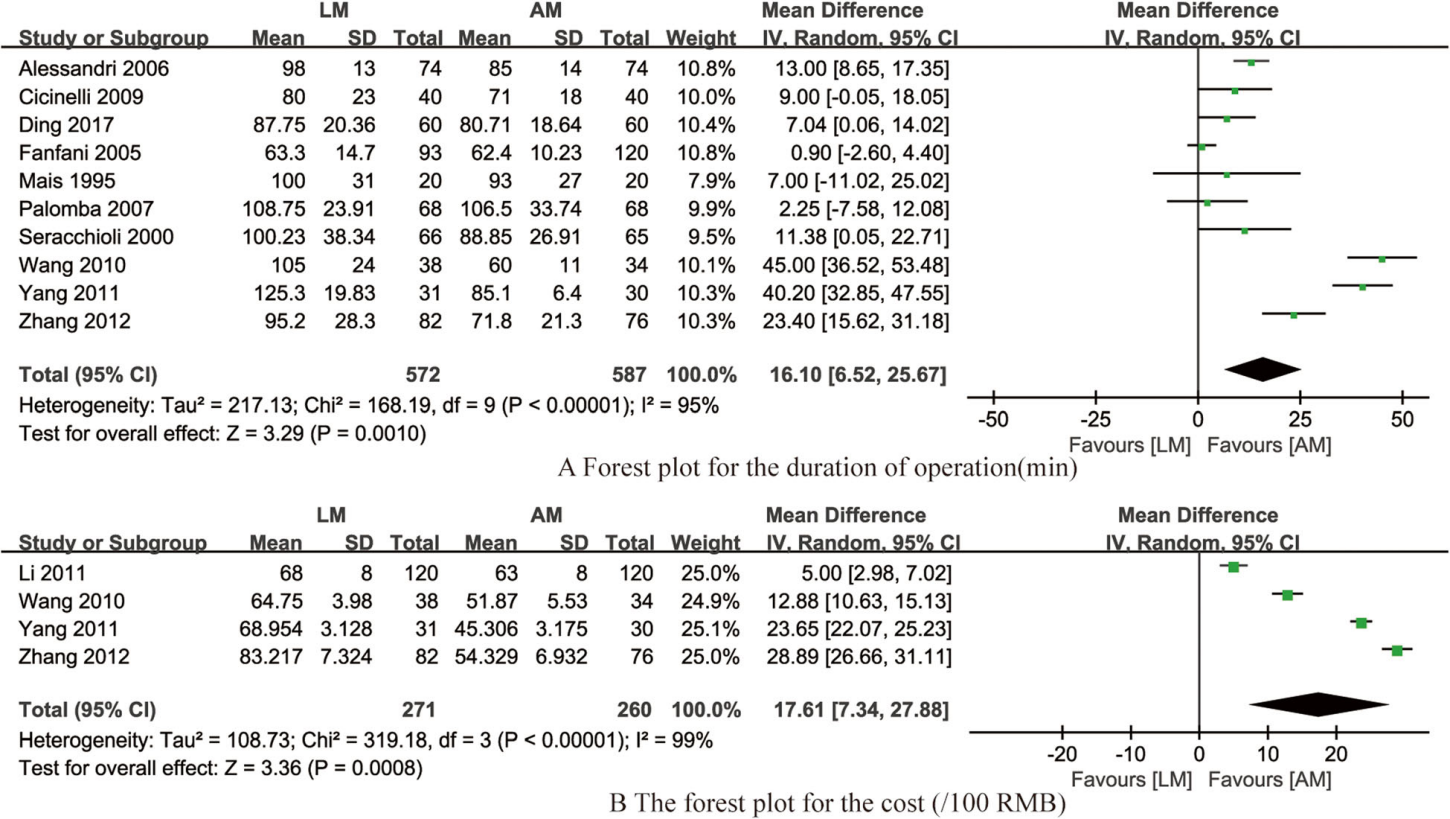
Forest plot for synthesized outcomes

Ten studies [13–16, 18–22, 24] reported the duration of operation, the pooled data from the ten RCTs revealed that LM was associated with longer duration of operation compared with AM (OR = 16.10, 95% CI 6.52–25.67, *P* < 0.001, *I*^*2*^ = 95%; Fig. 5a).

Medical cost Four studies [17, 21, 23, 24] reported the medical cost of LM and AM, the pooled data from the four RCTs revealed that LM was associated with higher medical cost compared with AM (OR = 17.61, 95% CI 7.34–27.88, *P* < 0.001, *I*^*2*^ = 99%; Fig. 5b).

### Subgroup and sensitivity analyses

**Fig. 6:**
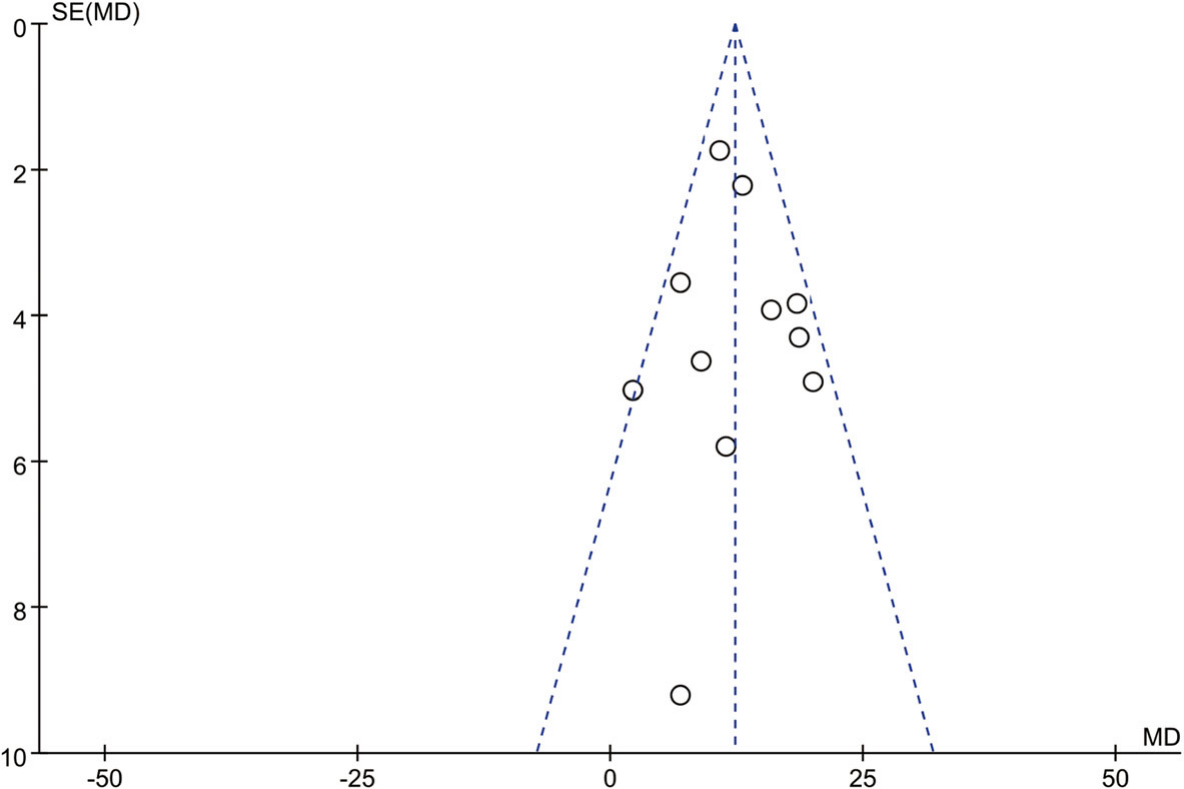
Funnel plot for the duration of operation

No subgroup analyses were performed in our study because the details of LM and AM of the included studies differed remarkably. We attempted to evaluate publication bias by using a funnel plot if 10 or more RCTs were included in an outcome meta-analysis (Fig. 6). Dots scattered symmetrically and evenly and no bias were found in the outcomes of duration of operation.

Sensitivity analyses, which investigate the influence of one study on the overall risk estimate by removing one study in each turn, suggested that the overall risk estimates were not substantially changed by any single study.

## Discussion

At present, the pathogenesis of uterine fibroids has not yet been fully understood [25]. The incidence of uterine fibroids in the childbearing age and perimenopausal period are higher, and the growth rate of fibroids is very fast [26]. However, the lesions of some patients after menopause will gradually shrink and even disappear [27]. Previous studies [28–30] have reported that the occurrence and development of multiple uterine fibroids is associated with changes of female hormones, the concentration of estradiol in myoma tissue is significantly higher than that of normal myometrial tissue, and the concentration of estrogen receptor is also significantly higher than the surrounding area of normal myometrial tissue, while the concentration of estradiol to estrone conversion is lower, it can be seen that progesterone, progesterone and its receptors can promote the occurrence and development of uterine fibroids. Although some patients with multiple uterine fibroids have small lesions and no obvious discomfort, and it’s likely to shrink or even disappear during perimenopausal period [31], more patients still have larger lesions and need surgery. LM and AM are common treatments for patients with multiple uterine fibroids, yet the effects and costs of LM and AM remain unclear. The results of our meta-analysis have revealed that LM can significantly reduce the blood loss, the duration of postoperative ileus and the length of hospital stay compared with AM, but it’s also associated with longer duration of operation and higher medical cost. To our knowledge, our study is the very rare meta-analysis to evaluate the role of LM and AM to provide a basis for clinical treatment.

Many previous studies [14, 32, 33] have found that LM for multiple uterine fibroids needs a longer operation time, and the intraoperative blood loss is significantly less than AM. Although the AM in uterine fibroids needs longer operation time, it can also significantly reduce the trauma. However, one included study [16] has found that the operation time of LM was similar to that of AM. The duration of the operation is affected by various factors such as anesthesia, the operation of the medical staff, and the proficiency of the surgeons [34–36], which may result in inconsistent results. However, theoretically the LM is more refined, and the requirements for the proficiency of clinicians are significantly higher [37], so it is understandable that LM needs longer operation time, but the surgery is small in trauma and can avoid larger surgical incisions in the abdomen, the blood loss can be significantly reduced accordingly.

Besides, the patients with LM for multiple uterine fibroids has a shorter postoperative hospital stay and fewer complications, indicating that the clinical application of LM can reduce related and complications and promote rapid restore. LM mainly uses the observation hole and the operation hole to understand the anatomical structure and the lesion, and completes the lesions removal under the guidance of the TV screen [38]. While the AM requires a long incision in the abdomen for direct vision, the trauma and the pulling force of related tissue during the AM are relatively large [39], hence the risk of complications is higher and the recovery is slower. It can be seen that the LM in patients with uterine fibroids has obvious advantages, which can promote the rapid recovery after surgery and enhance the safety of patients.

However, the medical cost cannot be ignored. To our knowledge, our meta-analysis is the first meta-analysis focused on the medical cost of LM and AM, which is different from previous related meta-analysis [40, 41]. On the one hand, the price of the surgical endoscope itself is relatively high, and the requirements for related auxiliary equipment in the LM process are relatively high [42], so the LM charge is relatively high. On the other hand, LM has higher requirements for surgeons, so the doctors performing LM have higher personal value requirements [43]. However, for hospitals that do not have endoscopic surgical conditions, AM may still be a better choice, with a relatively low price and relatively low proficiency requirements for surgeons. we should consider the actual situation clinically to choose LM and AM.

Several limitations in this study must be addressed. Firstly, the potential risk of bias in the allocation concealment process, blinding of researchers, blinding of outcome assessments or selective reporting must be considered, further studies with strict design are needed. Secondly, we identified high heterogeneity among the included trials, we attempted to conduct sub-group analysis to identify the source of heterogeneity, but the number and data information of included RCTs were limited, we couldn’t perform subgroup analysis. Finally, all the reported RCTs were from Italy and China, the population and area bias can exist, more related studies in different countries and populations are highlighted.

## Conclusions

In conclusion, LM may be more appropriate for patients with multiple uterine fibroids. Compared with AM, LM can significantly prolong the operation time and increase medical expenses, but it can also significantly reduce blood loss, shorten the duration of postoperative ileus and the length of hospital stay, the advantages are significantly more obvious. However, endoscopy should be preferred for the more favorable post-operative and clinical profile, but it should be avoided unprotected morcellation, as well as endoscopic approach with morcellation in women with suspicious leyomioma. Additional high-quality, large-scale multicenter RCTs are still warranted for identify the role of LM and AM in patients with uterine fibroids.

## Data Availability

All data generated or analyzed during this study are included in this published article.

## Abbreviations

AM: Abdominal myomectomy
CI: Confidence interval
LM: Laparoscopic myomectomy
MD: Mean differences
OR: Odd ratios
RCTs: Randomized controlled trials
